# Antioxidant Activity and Cytotoxicity of *Medicago sativa* L. Seeds and Herb Extract on Skin Cells

**DOI:** 10.1089/biores.2020.0015

**Published:** 2020-10-23

**Authors:** Martyna Zagórska-Dziok, Aleksandra Ziemlewska, Zofia Nizioł-Łukaszewska, Tomasz Bujak

**Affiliations:** Department of Technology of Cosmetic and Pharmaceutical Products, University of Information Technology and Management in Rzeszow, Tyczyn, Poland.

**Keywords:** antioxidants, free radicals, cell culture, oxidative stress

## Abstract

In response to the constantly growing demand for high quality cosmetics we observe an increasing interest in products containing natural plant extracts. This article attempts to assess the antioxidant and cytotoxic properties of extracts from alfalfa herb and seeds (*Medicago sativa* L.). These extracts were obtained using ultrasound-assisted extraction method. The research was carried out on two cell lines: fibroblasts and keratinocytes. The obtained results show that the examined extracts from *M. sativa* L. are a source of valuable biologically active agents. Both extracts were characterized by high content of flavonoids and phenolic compounds. Evaluation of antioxidant properties of extracts using DPPH• radical indicated that the alfalfa extracts can efficiently scavenge free radicals. The results of the conducted experiments demonstrated that the *M. sativa* L. extracts do not only have an inhibitory effect on free radicals existing in the external environment of the cell, but also have the ability to reduce the intracellular reactive oxygen species level, which may contribute to the reduction of oxidative stress inside the cells. Studies performed using cell lines indicated that the tested extracts increase the proliferation and metabolism of skin cells *in vitro*. The high antioxidant capacity of *M. sativa* L. extracts may indicate its potential use as a valuable ingredient in the cosmetics and pharmaceutical industries.

## Introduction

The skin is a barrier that separates the body from the external environment. In addition to protecting the body against water loss and infection of microorganisms, it plays an important cosmetic role.^1^ As the most extensive organ in the body, over time the skin shows numerous visible signs of aging. Therefore, many people, especially women, spend a lot of money on cosmetics and pharmaceutical products that help them prevent or partially reverse skin aging.^2^ This aging is caused by both internal and external factors. Internal aging is an unavoidable physiological process that results in thin, fine wrinkles, dry skin, and progressive skin atrophy. In contrast, external aging is caused by external factors such as air pollution, poor nutrition, smoking, and exposure to the sun, which leads to thick wrinkles, laxity and loss of skin elasticity, and rough textured appearance. Moreover long-term exposure to solar ultraviolet (UV) radiation is the basic factor of skin aging called photoaging.^3,4^

As is commonly known, skin aging is affected by reactive oxygen species (ROS) such as superoxide anions (O_2_·^−^), hydroxyl, nitric oxide radicals, and hydrogen peroxide (H_2_O_2_). They play an important role in oxidative stress associated with the pathogenesis of various diseases.^5^ Excessive production of free radicals may cause damage to cells and tissues through nonspecific modification and disruption of proteins, phospholipids, and nucleic acids. ROS have a serious negative effect on organelles and cell membranes. Their excess causes peroxidation of membrane lipids and modification of membrane proteins. As a result, the membrane structure is changing and their functions are disturbed.^6,7^

Fortunately, well-functioning cells have the ability to defend against the destructive effects of free radicals by the endogenous systems consisting of various enzymes, such as catalase, superoxide dismutase, and glutathione peroxidase. Many scientists have shown that it is possible to reduce the negative influence of free radicals by using appropriate protective agents, that is, antioxidants. Natural antioxidants may be a useful strategy for the prevention of photoaging and oxidative stress closely related to skin diseases.^8–11^ The rich source of natural antioxidants are plants, mainly vegetables, fruits, and herbs, which are a common component of the daily diet.^12^ Plants extracts are currently one of the largest and most valued groups of ingredients used in the pharmaceutical and cosmetics industries. In addition to their strong antibacterial, antifungal, and antiviral activity, they also show strong antioxidant properties.^13,14^ The antioxidant capacity of plant extracts is mainly related to the content of phenols, flavonoids, isoflavonoids, and anthocyanins. Numerous studies have shown a correlation between the content of phenolic compounds and flavonoids in plants and the antioxidant activity of plant extracts.^15,16^ Plant extracts are used in cosmetics mainly to improve their quality and ameliorate functionality. It has been shown that the extracts are much more effective than single compounds due to synergistic interactions between all chemical components.^17,18^ One of the plants containing abundant active ingredients that can be extracted and used as active ingredients in cosmetics preparations is *Medicago sativa* L.

*M. sativa* L. known as alfalfa is a member of the Fabaceae family, which has a long dietary and medicinal use in traditional herbal medicine in China, India, America, and many Middle Eastern countries for the treatment of a variety of disorders.^19^ In addition, *M. sativa* L. is widely used as animal feed due to the high content of fiber, proteins, minerals, vitamins, chlorophyll, and carotenoids.^20^ It has also been documented that alfalfa is a valuable source of phytochemicals such as alkaloids, amino acids, carotenes, coumarins, digestive enzymes, flavonoids, minerals, nonprotein amino acids, organic acids, phenolic compounds, cyclic polyols, phytoestrogens, phytosterols, polyamines, saponins, and some other volatile organic compounds such as terpenes, furanoids, alcohols, and ketones. The impressive amount of biologically active compounds certainly contributed to the fact that this plant has antioxidant, anti-inflammatory, immunomodulatory, and anticancer properties.^21–23^

The aim of our research was to assess the effect of seed and herb alfalfa extracts on the viability of skin cells, fibroblasts (BJ) and keratinocytes (HaCaT) *in vitro*. These cells were selected because this article is an attempt to assess the potential use of extracts from this plant in pharmaceutical and cosmetic preparations. Due to the fact that oxidative stress plays an extremely important role in skin condition, this work also includes an assessment of the ability to scavenge free radicals, both in the external environment and inside the cells.

## Materials and Methods

### Plant material and extraction procedure

Seeds and herb of *M. sativa* L. were obtained from the local herbalist. The Plato variety was used in the research. It was produced by Diet-Food, Poland. Then two extracts were made, separately from the herb and alfalfa seeds.

### Ultrasound-assisted extraction method

Extracts from alfaalfa seeds and herb were obtained using ultrasound-assisted extraction method (UAE). UAE was performed according to the method described by Yang et al. in ultrasonic bath (Digital Ultrasonic Cleaner) equipped with time controller.^24^ About 20 g of *M. sativa* L. seeds and herb were placed into a glass beaker and 180 mL a mixture of water and glycerine in the ratio of 80:20 was poured. The mixture was homogenized in room temperature for 50 min (10 cycles for 5 min). Then, obtained extracts were collected and filtered three times through Whatman filter paper No. 10 using a vacuum pump. Extracts were stored in the dark in 4°C for further analysis.

### Total phenolic content determination

The total phenolic content in extracts obtained from alfalfa seeds and herb were determined spectrophotometrically by the Folin-Ciocalteu method according to the procedure reported by Singleton et al. with some modifications.^25^ The 300 μL of tested extract (0.25–10%) and 1500 μL of 1:10 Folin-Ciocalteau reagent were mixed. After 6 min of incubation in the dark, 1200 μL of sodium carbonate (7.5%) was added to each sample. After 2 h of incubation in the dark the absorbance of the tested solutions was measured spectrophotometrically at λ = 740 nm by AquamateHelion (Thermo Scientific). The measurements were carried out at room temperature (25°C). The total phenolic concentration in analyzed extracts were calculated from a gallic acid (GA) calibration curve (10–100 mg/mL). The estimation of phenolic compounds in the extracts was carried out in triplicate and the results were averaged. 

### Total flavonoids content determination

The total flavonoid content (TFC) of analyzed extracts were evaluated using aluminium nitrate nonahydrate according to the procedure reported by Matejić et al. with modifications.^26^ Briefly, 600 μL of examined extract solutions (0.25–10%) and 2400 μL of mixture (80% C_2_H_5_OH, 10% Al(NO_3_)_3_ × 9 H_2_O and 1 M C_2_H_3_KO_2_) were mixed. After 40 min of incubation at 25°C, the absorbance at 415 nm was measured spectrophotometrically by FilterMax F5 (AquamateHelion). Both types of extracts were used in the analyzes. The total flavonoids concentration in *M. sativa* L. extracts were calculated from a quercetin hydrate (Qu) calibration curve (10–100 mg/mL) and expressed as quercetin equivalents (Qu)/g of extract averaged from three independent measurements.

### DPPH Radical Scavenging Assay

The ability to scavenge free radicals by alfalfa extracts obtained from seed and herb was evaluated using DPPH (2,2-diphenyl-1-picrylhydrazyl) assay according to the method described by Brand-Williams et al.^27^ About 167 μL of 4 mM ethanol solution of DPPH was mixed with 33 μL of analyzed samples in different concentrations (0.25%, 0.5%, 1%, 2.5%, 5%, and 10%). The absorbance was measured at λ = 516 nm in every 5 min for 30 min. Measurements were made using UV-Vis spectrophotometer Filter Max 5 (Thermo Scientific). DPPH solution mixed with equal volume of distilled water was used as a control. The percentage of DPPH radical scavenging was calculated using the equation:

%DPPH∙scavenging=Abs control−Abs sampleAbs control×100%

### Cell culture

BJ cells (fibroblasts, ATCC^®^CRL-2522™) and HaCaT cells (normal human keratinocytes, ATCC) used in the experiments were obtained from the American Type Culture Collection (Manassas, VA). Cells were maintained in a Dulbecco's modified essential medium (DMEM; Gibco) with l-glutamine, supplemented with 10% (vol/vol) fetal bovine serum (FBS; Gibco), and 1% (vol/vol) antibiotics (100 U/mL Penicillin and 1000 μg/mL Streptomycin; Gibco). Fibroblasts were maintained in a Minimum Essential Medium (MEM; Gibco) that contains Earle's salt and l-glutamine, supplemented with 10% (vol/vol) FBS (Gibco), and 1% (vol/vol) antibiotics (100 U/mL Penicillin and 1000 μg/mL Streptomycin; Gibco). The cultured cells were kept at 37°C in a humidified atmosphere of 95% air and 5% of carbon dioxide. When the cells reached confluence, the culture medium was removed from the flask (Nest) and cells were rinsed two times with sterile phosphate-buffered saline (PBS; Gibco). The confluent layer was trypsinized using Trypsin/EDTA (Gibco) and then resuspended in fresh medium. The cells were seeded in a 96-well plate bottom (on separate plates for each cell type). After attachment of the HaCaT and fibroblasts to the bottom of the wells of the plates, cells were incubated with varying concentrations (0.25%, 0.5%, 1%, 2.5%, 5%, and 10%) of the aqueous glycerin extracts from *M. sativa* L. seeds and herb.

### Cell viability assay

#### Neutral red uptake assay

The neutral red uptake assay (Sigma Aldrich) was used to assess HaCaT and fibroblasts viability. This assay is based on the initial protocol described by Borenfreund and Puerner.^28^ It allows to evaluate cell viability and determine accumulation of the neutral red dye in the lysosomes of viable, uninjured cells. The examined cells were placed in 96-well plates at a density of 1 × 10^4^ cells/well. After 24 h of preculture, medium (DMEM or MEM) was aspirated and tested concentrations of *M. sativa* L. UAE seeds and herb extracts (in the range of concentrations from 0.25% to 10%) were added into each well and cultured for another 24 h. The control group was unexposed cells. Following exposure to tested extracts, cells were incubated for 2 h with neutral red dye (40 μg/mL) dissolved in serum-free DMEM or MEM medium. Then, cells were washed with PBS and 150 μL of destain solution (EtOH/AcCOOH/H_2_O_2_, 50%/1%/49%) per well was added. The plates were shaken gently for 10 min until the neutral red had been extracted from the cells. Neutral red dye uptake was determined by measuring the optical density of the eluted dye at λ = 540 nm in microtiter plate reader spectrophotometer FilterMax F5 (Thermo Fisher). The experiments were performed in triplicates for each extract concentration and presented as percentage of control values (100%).

#### Alamar Blue assay

Another test to assess cell viability was the Alamar Blue test (resazurin sodium salt, Sigma, R7017). Cells were seeded in transparent 96-well plates at a density of 1 × 10^4^ cells/well with fresh DMEM or MEM medium and exposed to different concentrations (0.25–10%) of tested alfalfa seeds and herb extracts for 24 h. The control group was unexposed cells maintained in a DMEM or a MEM medium. After 24 h of exposure, resazurin solution was transferred into the plates for a final volume of 250 μL/well and final concentration of 60 μM resazurin. Subsequently, the cells were incubated for 2 h at 37°C in darkness. The absorbance was measured at the wavelength λ = 570 nm using a microplate reader (FilterMax F5; Molecular Devices). The experiments were performed in triplicates for *each M. sativa* L. extracts concentration. Results were expressed as a percentage of the viability of the control sample versus the control (100%).

### Detection of intracellular ROS level

To assess the ability of the analyzed *M. sativa* L. extracts to change the intracellular level of ROS in HaCaT and fibroblast cells, the fluorogenic dye H_2_DCFDA was applied. Analogous to the tests evaluating cell viability, the analyzed cells were seeded in 96-well plates at a density of 1 × 10^4^ cells per well and initially cultured before the experiment for 24 h. In the next step, the culture medium was removed and 10 μM H_2_DCFDA (Sigma Aldrich) in serum-free medium (DMEM or MEM for HaCaT and fibroblasts, respectively) was added. Cells were incubated with H_2_DCFDA for 45 min before extracts treatment. Subsequently, HaCaT and fibroblast cells were exposed into different glycerin-water extract concentrations (0.25%, 0.5%, 1%, 2.5%, 5%, and 10%). Cells treated with 1 mM H_2_O_2_ were used as positive control. The 2′,7′-dichlorofluorescein (DCF) fluorescence was monitored every 30 min for a total 90 min using a microplate reader FilterMax F5 (Thermo Fisher) at maximum excitation of λ = 485 nm and emission spectra of λ = 530 nm.

### Statistical analysis

Obtained values were presented as a mean ± SD. Significant differences between obtained values were analyzed using StatSoft, Statistica 9.0 using one-way analysis of variance and Tukey's test. Differences were considered significant when *p* < 0.05. Statistically significant differences are marked on the charts with letters. Statistically significant differences were marked with different letters.

## Results

The presence of only a few studies comparing active compounds in different parts of alfalfa contributed to the undertaking of studies aimed at assessing the antioxidant properties of water-glycerin extracts from seeds and herbs of *M. sativa* L. The study also determined the biological activity of these extracts using *in vitro* model cell lines.

The article attempts to determine the total content of phenols (TPC) and the TFC. The amounts of these compounds were determined from calibration curves of gallic acid (*y* = 0.0046x + 0.0452, R2 = 0.9989), and quercetin (*y* = 0.0153x − 0.0053, R2 = 0.9996), respectively. The analysis was carried out for six different dilutions (0.25%, 0.5%, 1%, 2.5%, 5%, and 10%) of each extract. The obtained results show that the highest amount of phenols and flavonoids was demonstrated in the 10% water-glycerine herb extract. The lowest concentration of these compounds showed a water-glycerine extract obtained from *M. sativa* L. seeds at a concentration of 0.25%. Similar trends were observed for the flavonoid content. The difference between the content of phenolic compounds in the herb compared to seeds at the highest concentration of 10% was around 90% and in concentration of 5% it was around 75%. Taking into account the content of flavonoids the difference between the herb and seeds was around 15% in case of concentration of 10% and around 35% in concentration of 5%. It was also observed that TPC and TFC increased in a dose-dependent manner for all the extracts tested (Table 1).

**Table 1.** Total Phenol Content and Total Flavonoids Content of Various Concentrations of Water-Glycerine Extracts of *Medicago sativa* L.

	TPC [mg GA/g]		TFC [mg Qu/g]	
Extract concentration [%]	Seeds	Herb	Seeds	Herb
0.25	2.48 ± 0.22^a^	3.52 ± 0.56^b^	0.65 ± 0.05^c^	1.58 ± 0.40^d^
0.5	4.43 ± 0.48^b^	9.46 ± 0.39^e^	1.36 ± 0.04^d^	4.12 ± 0.23^b^
1.0	8.67 ± 0.29^g^	12.58 ± 0.4^h^	3.55 ± 0.43^b^	5.33 ± 0.47^f^
2.5	11.55 ± 0.37^h^	19.84 ± 0.26^j^	5.52 ± 0.41^f^	9.47 ± 0.42^g^
5.0	20.58 ± 0.36^j^	36.62 ± 0.43^k^	13.87 ± 0.11^i^	20.84 ± 0.71^j^
10.0	37.60 ± 1.82^k^	73.5 ± 0.53^l^	21.35 ± 0.63^j^	43.38 ± 0.65^m^

Values are mean of three replicate determinations (*n* = 3) ± SD. Values not sharing the same letter are significantly different at *p* < 0.05.

GA, gallic acid; TFC, total flavonoid content; TPC, total phenol content.

The next stage of our research was the assessment of the ability of extracts from seeds and herb to remove free radicals. Therefore, the antioxidant potential was assessed using the DPPH radical. To determine the antioxidant properties of the extracts tested, six different concentrations were used spanning from 0.25% to 10%. Measurements were taken every 5 min in 30 min. Based on the obtained data, it was shown that each extract has a different ability to reduce free radicals.

**FIG. 1.**
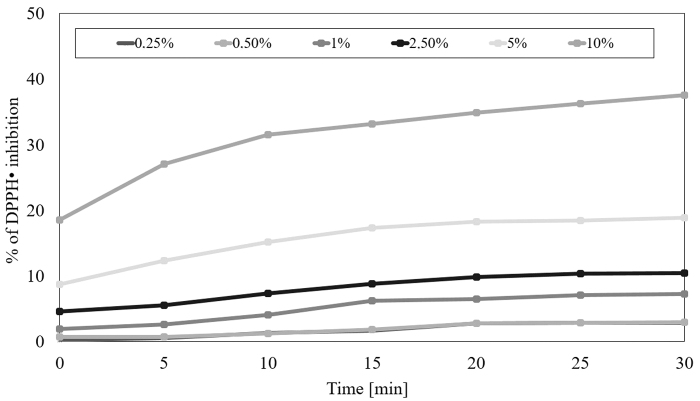
Kinetics of the absorbance changes in DPPH· solutions in the presence of various concentrations of water-glycerine extract of *Medicago sativa* L. seeds. Values are mean of three replicate determinations (*n* = 3). DPPH, 2,2-diphenyl-1-picrylhydrazyl.

**FIG. 2.**
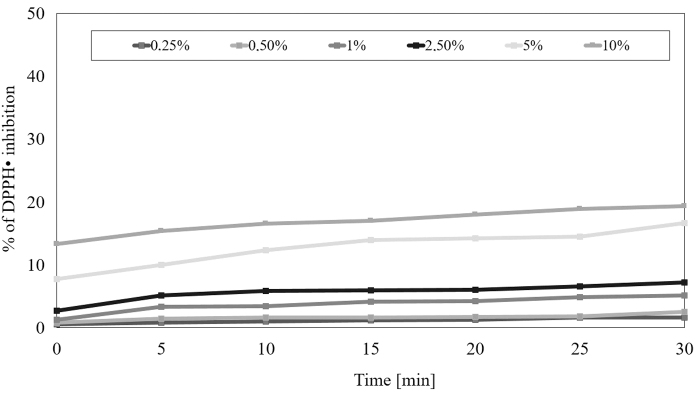
Kinetics of the absorbance changes in DPPH· solutions in the presence of various concentrations of water-glycerine extract of *M. sativa* L. herb. Values are mean of three replicate determinations (*n* = 3).

The highest ability of the DPPH radical scavenging was shown in the water-glycerine extract from the seeds at the highest concentration tested equal 10%. After 30 min of the test the level of reduced DPPH• reached almost 40%, while at the lowest concentration of 0.25% the level of scavenged radicals was about 3%. In the case of herb, the level of scavenging of free radicals was significantly lower than in case of seeds in all concentrations tested, and at the highest concentration tested equal 10% and after 30 min it was about 20%. In both analyzed raw materials, a correlation between the concentration and antioxidant potential of the extracts was also observed—higher the concentration used higher the force of free radicals reduction (Figs. 1 and 2).

**FIG. 3.**
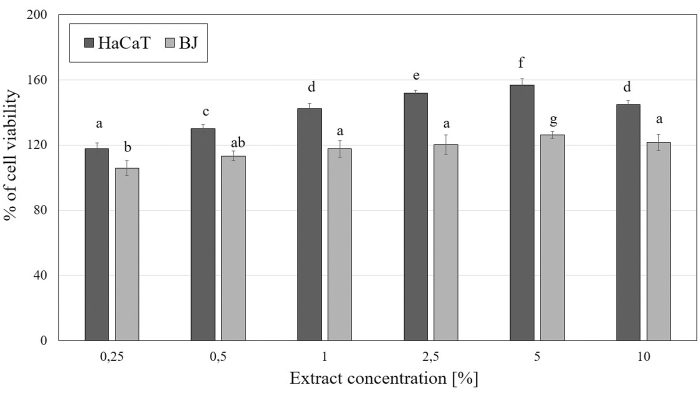
The effect of increasing concentrations of *M. sativa* L. seeds extract (0.25–10%) on Neutral Red Dye uptake in cultured keratinocytes (HaCaT) and fibroblasts (BJ) after 24 h of exposure. Data are the mean ± SD of three independent experiments, each of which consists of three replicates per treatment group. Values not sharing the same letter are significantly different at *p* < 0.05.

**FIG. 4.**
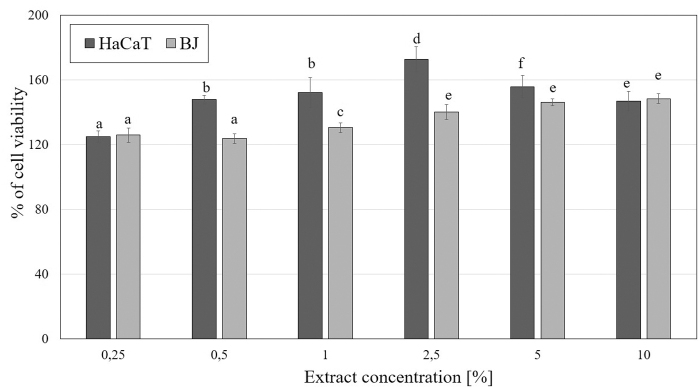
The effect of increasing concentrations of *M. sativa* L. herb extract (0.25–10%) on Neutral Red Dye uptake in cultured keratinocytes (HaCaT) and fibroblasts (BJ) after 24 h of exposure. Data are the mean ± SD of three independent experiments, each of which consists of three replicates per treatment group. Values not sharing the same letter are significantly different at *p* < 0.05.

An extremely important aspect in assessing the safety and toxicity of plant extracts is to evaluate their impact on cell metabolism and viability. In this study, we focused on the evaluation of cytotoxic properties of *M. sativa* L. extracts on two cell lines—fibroblasts (BJ) and keratinocytes (HaCaT). The tests used in the study (Neutral Red assay and Alamar Blue assay) allow to assess the degree of inhibition of growth related to concentration of the examined *M. sativa* L. extracts. The results of the conducted experiments using Neutral Red assay indicate the positive effect of alfalfa extracts on the viability of both analyzed cell lines. This effect differs between fibroblasts and keratinocytes and is closely related to the concentration used. It was observed that these extracts exert a stronger effect on the viability of HaCaT than BJ cells, resulting in a greater increase in viability of these cells. The studies also showed that a slightly better effect is caused by the extract obtained from alfalfa herb in comparison with the seed extract. Alfalfa herb extract caused a slightly greater increase in keratinocytes viability to over 170% (at a concentration of 2.5%), whereas in the case of seed extract this value reached less than 160% (at a concentration of 5%). In the case of fibroblasts, the use of the highest concentrations of *M. sativa* L. herb extract resulted in an increase in fibroblast viability to about 150%, while the seed extract increased the proliferation of these cells to less than 130% (at a concentration of 5%) (Figs. 3 and 4).

**FIG. 5.**
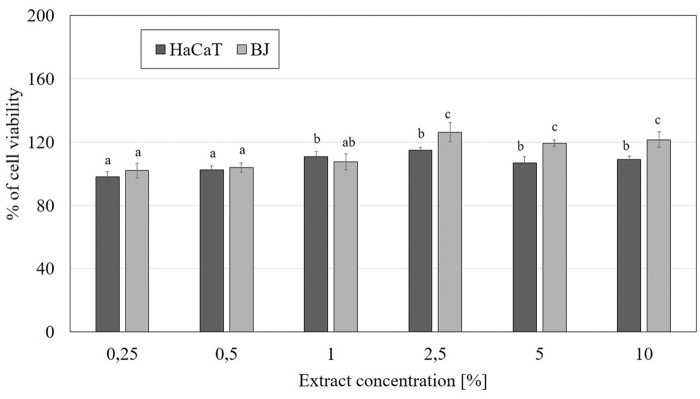
The reduction of resazurin after 24 h exposure to the *M. sativa* L. seeds extract (0.25–10%) in cultured keratinocytes (HaCaT) and fibroblasts (BJ). Data are the mean ± SD of three independent experiments, each of which consists of three replicates per treatment group. Values not sharing the same letter are significantly different at *p* < 0.05.

**FIG. 6.**
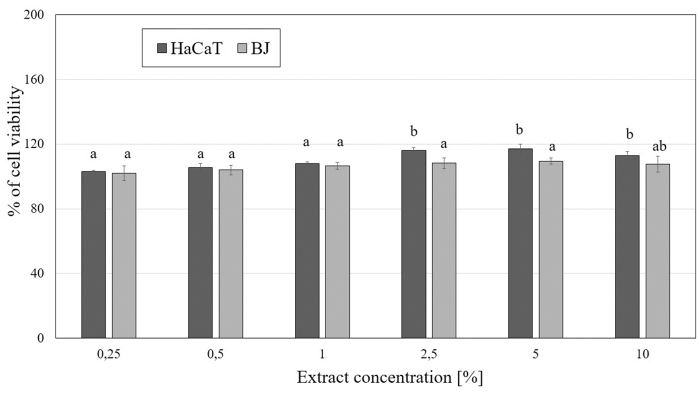
The reduction of resazurin after 24 h exposure to the *M. sativa* L. herb extract (0.25–10%) in cultured keratinocytes (HaCaT) and fibroblasts (BJ). Data are the mean ± SD of three independent experiments, each of which consists of three replicates per treatment group. Values not sharing the same letter are significantly different at *p* < 0.05.

Similar results were obtained using the Alamar Blue assay. The amount of resazurin reduced by the examined cells allowed a quantitative assessment of cell viability. This test also demonstrated that both alfalfa seeds and herb UAE extracts have a positive effect on the viability of both cell lines. However, the differences observed between fibroblasts and keratinocytes were lower than those observed on the first test. The results of the analyzes performed with the use of this assay showed that the seeds extract strongly stimulates the viability of fibroblasts than keratinocytes and this increase reaches up to 130% (in the case of the three highest concentrations analyzed). Incubation of cells with extracts obtained from alfalfa herb resulted in an increase in HaCaT cells viability up to 120%, whereas in the case of BJ cells no significant increase in viability was observed (Figs. 5 and 6).

**FIG. 7.**
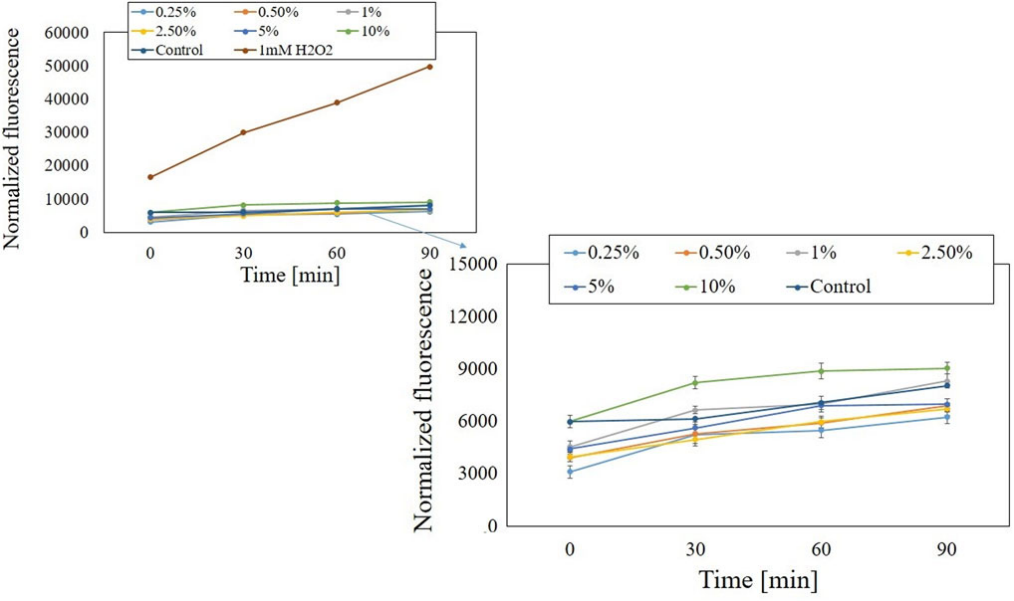
The effect of increasing concentrations of water-glycerine *M. sativa* L. seeds extract (0.25–10%) on the DCF fluorescence in HaCaT cells. Medium with 1 mM H_2_O_2_ was used as a positive control. The data are expressed as the mean ± SD of three independent experiments, each of which consisted of three replicates per treatment group. DCF, 2′,7′-dichlorofluorescein; H_2_O_2_, hydrogen peroxide.

**FIG. 8.**
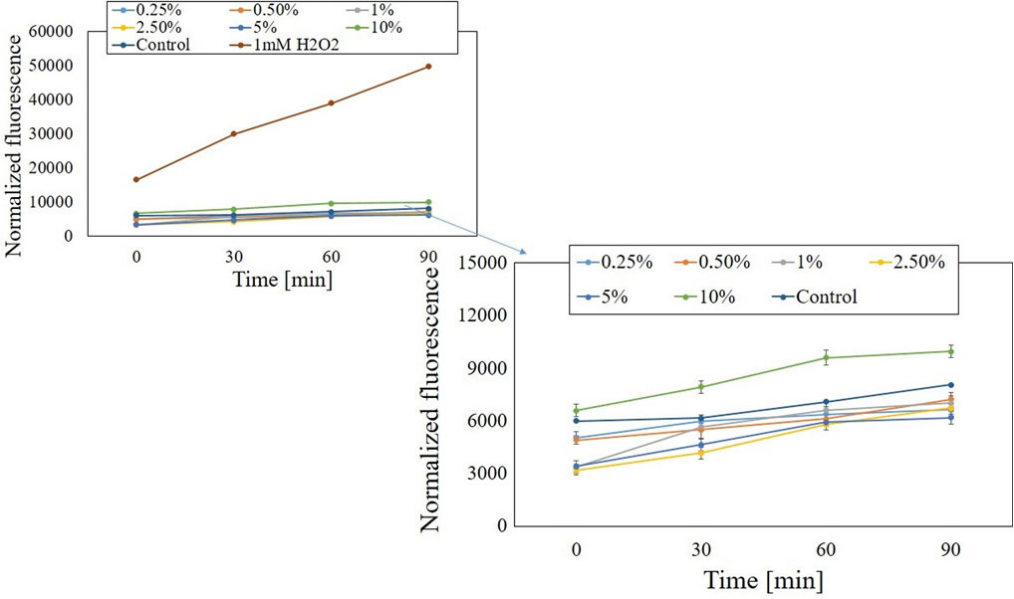
The effect of increasing concentrations of water-glycerine *M. sativa* L. herb extract (0.25–10%) on the DCF fluorescence in HaCaT cells. Medium with 1 mM H_2_O_2_ was used as a positive control. The data are expressed as the mean ± SD of three independent experiments, each of which consisted of three replicates per treatment group.

To detect a disturbed redox balance in examined cells after exposure to *M. sativa* L. extracts the fluorogenic dye H_2_DCFDA was applied. This is one of the most commonly used probe for detecting intracellular ROS and oxidative stress.^29^ Before starting measurements using cell lines, we examined whether seeds and herb alfalfa UAE extracts without cells affected the fluorescence of the H_2_DCFDA. Additionally, the separated experiment showed that there were no interactions between examined extracts and H_2_DCFDA substrate in DMEM and MEM medium. The tests carried out showed that examined extracts can generate intracellular ROS in time- and dose-dependent manner. The activity of tested *M. sativa* L. extracts was also specific to the cell model. HaCaT cells treated with extracts in the concentration range from 0.25% to 10% exhibited correlation between used dose and intracellular free ROS level. After incubation of these cells with individual concentrations of extracts (both from the herb and seeds), an increase in the number of ROS was noted only at the highest concentration used (10%). When alfalfa extracts were used in concentrations from 0.25% to 5%, a decrease in intracellular ROS level was observed for both types of extracts (Figs. 7 and 8).

**FIG. 9.**
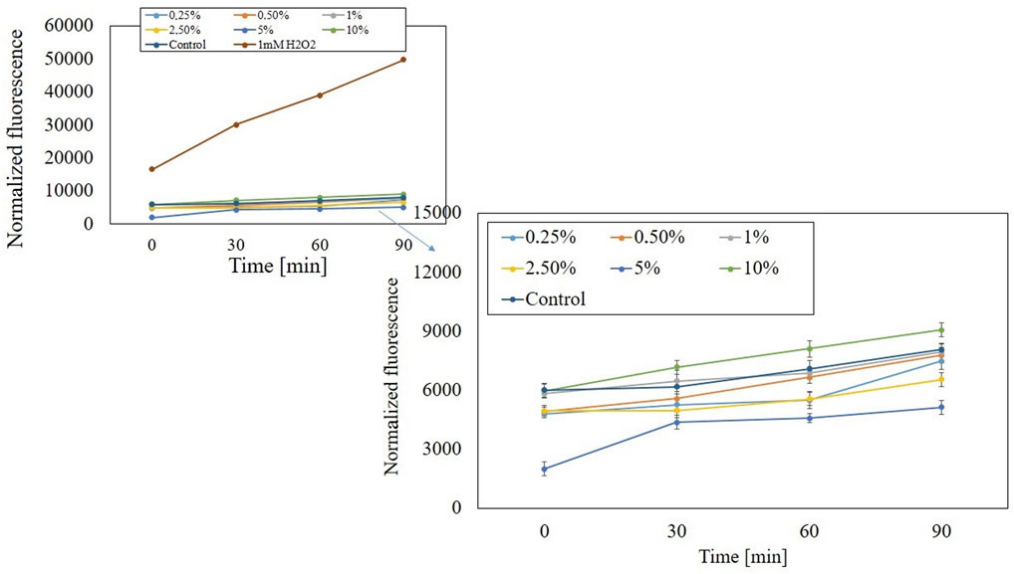
The effect of increasing concentrations of water-glycerine *M. sativa* L. seed extract (0.25–10%) on the DCF fluorescence in BJ cells. Medium with 1 mM H_2_O_2_ was used as a positive control. The data are expressed as the mean ± SD of three independent experiments, each of which consisted of three replicates per treatment group.

**FIG. 10.**
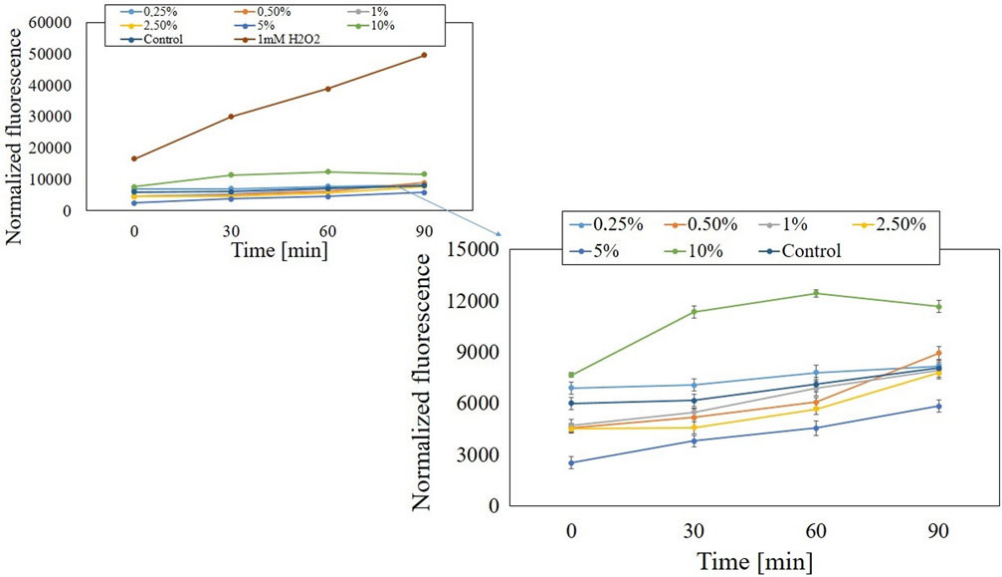
The effect of increasing concentrations of water-glycerine *M. sativa* L. herb extract (0.25–10%) on the DCF fluorescence in BJ cells. Medium with 1 mM H_2_O_2_ was used as a positive control. The data are expressed as the mean ± SD of three independent experiments, each of which consisted of three replicates per treatment group.

Analogous results were obtained when measuring the intracellular level of ROS in fibroblasts. As in the case of keratinocytes, the highest concentration of analyzed extracts increased the level of ROS. The values obtained after exposure of BJ cells to lower concentrations of both types of extracts fluctuated below the value obtained for the control (cells not exposed to the extract) or were significantly lower, which may indicate a decrease in intracellular ROS production significantly. Only after the fibroblasts were treated with the water-glycerine extracts of *M. sativa* L. herb at a concentration of 0.25%, the level of ROS oscillated slightly above the value obtained for the control (Figs. 9 and 10).

## Discussion

Plant raw materials, with a large amount of biologically active substances can affect the activity and metabolism of cells. The main group of such substances includes polyphenolic compounds, which are one of the functional plant substances. They are characterized by strong antioxidant, anti-inflammatory, antifungal, and antiallergic properties.^30–32^ Chemical structure, type, and a level of the oxidation of the substrate and the conditions under which the oxidation process took place influence their activity.^22,33^ Both seeds and leaves are rich in compounds having antioxidant properties such as kaempferol, quercetin, luteolin, silbenes, coumarins, and others.^34,35^

The experiments carried out in this article clearly showed that the extract of alfalfa herb is a much richer source of biologically active substances compared with the extract obtained from the seeds of this plant. Thus, the herbal extract shows greater potential and may be beneficial in many aspects.

As mentioned above, the essential properties of phenolic compounds derived from plant material are their antioxidant properties. These compounds consist of a hydroxyl group and play a major role in the antioxidant capacity thanks to hydrogen releasing and the formation of stable radical intermediates.^32^ The mechanism of their action consists mainly of neutralization of free radicals, enzyme induction, and chelation of metal ions.^21,36^

The results of the DPPH• scavenging test obtained in this article correspond with other studies. Kudan and Anupam showed strong antioxidant activity of extracts obtained from *M. sativa* L.^35^ High antioxidant activity was caused by a significant content of phenolic compounds, in particular quercetin or kaempferol. According to Rana et al. the extracts obtained from the roots of *M. sativa* L also show strong antioxidant activity.^22^

The cytotoxicity studies carried out in the next stage using the Alamar Blue and Neutral Red assays showed a beneficial effect of alfalfa extracts on the analyzed skin cells, both HaCaT and BJ. Although the results of both tests differ slightly, both clearly indicate that the extracts, both from the herb and seeds of *M. sativa* L., have a positive effect on the viability of fibroblasts and keratinocytes. The results obtained in these studies for the first time indicate the lack of toxicity of alfalfa extracts against skin cells and may suggest the potential use of these extracts in the pharmacological, dermatological, and cosmetic industries. In addition, the extracts obtained from this plant can be helpful in the fight against cancer, because experiments carried out by other researchers indicate their cytotoxic effects on several sensitive and multidrug-resistant tumor cells lines.^37^

As mentioned above, there are many articles indicating the antioxidant properties of alfalfa.^21,22,35,36^ As part of this work, these properties were also confirmed for the herb and seeds extracts of *M. sativa* L. However, until now, there have been no studies assessing the effect of alfalfa extracts on the level of ROS inside cells treated with these extracts. Thus, the results of the conducted experiments indicate that the *M. sativa* L. extracts not only affect the scavenging of free radicals existing in the external environment of the cell, but also have the ability to reduce the intracellular ROS level, which may contribute to the reduction of oxidative stress inside the cells. Due to the fact that oxidative stress leads to numerous intracellular disorders^38^ the exposure of skin cells to alfalfa extracts can have a positive effect.

## Conclusion

The article was an attempt to determine the properties of extracts from seeds and herb of *M. sativa* L. The tested extracts were characterized by a high content of biologically active phenolic compounds and flavonoids. It has been shown that the extracts have an antioxidant potential. It was also noted that tested extracts have a positive effect on the viability and proliferation of skin cells. The beneficial properties of alfalfa extracts shown in this article indicate the potential possibility of their use in the production of various types of cosmetic and pharmaceutical preparations applied to the skin. Although preliminary *in vitro* cytotoxicity studies on cells that are part of the various layers of the skin indicate no cytotoxicity, there is a need for further *in vivo* studies using animal models and clinical trials. Conducting this type of research will allow to assess the full safety of *M. sativa* L. extracts, which show promising properties in *in vitro* conditions.

## Abbreviations Used

DCF: 2′,7′-dichlorofluorescein
DMEM: Dulbecco's modified essential medium
DPPH: 2,2-diphenyl-1-picrylhydrazyl
FBS: fetal bovine serum
GA: gallic acid
H_2_O_2_: hydrogen peroxide
MEM: Minimum Essential Medium
PBS: phosphate-buffered saline
ROS: reactive oxygen species
TFC: total flavonoid content
TPC: total phenol content
UAE: ultrasound-assisted extraction method
UV: ultraviolet

## References

[1] Blanpain C, Fuchs E Epidermal stem cells of the skin. Annu Rev Cell Dev Biol. 2006;22:339–373 16824012 10.1146/annurev.cellbio.22.010305.104357 PMC2405915

[2] Kazanci A, Kurus M, Atasever A Analyses of changes on skin by aging. Skin Res Technol. 2016;23:48–60 27321201 10.1111/srt.12300

[3] Krutmann J, Bouloc A, Sore G, et al. The skin aging exposome. J Dermatol Sci. 2017;85:152–161 27720464 10.1016/j.jdermsci.2016.09.015

[4] Mora Huertas AC, Schmelzer CE, Hohenwarter W, et al. Molecular-level insights into aging processes of skin elastin. Biochimie. 2016;128–129:163–173 10.1016/j.biochi.2016.08.010 27569260

[5] Devasagayam TPA, Tilak JC, Boloor KK, et al. Review: free radicals and antioxidants in human health: current status and future prospects. J Assoc Phys India. 2004;52:794–804 15909857

[6] Hunt JV, Dean RT, Wolff SP Hydroxyl radical production and autoxidative glycosylation. Glucose autoxidation as the cause of protein damage in the experimental glycation model of diabetes mellitus and ageing. Biochem J. 1988;256:205–212 2851978 10.1042/bj2560205 PMC1135388

[7] Casaril M, Corso F, Corrocher R Free radicals in human pathology. Recenti Prog Med. 1991;82:39–44 2028076

[8] Kancheva VD, Kasaikina OT Bio-antioxidants—a chemical base of their antioxidant activity and beneficial effect on human health. Curr Med Chem. 2013;20:784–805 10.2174/09298673113209990161 24274817

[9] Nimse SB, Pal D Free radicals, natural antioxidants, and their reaction mechanisms. RSC Adv. 2015;5:27986

[10] Lü JM., Lin PH, Yao Q, et al. Chemical and molecular mechanisms of antioxidants: experimental approaches and model systems. J Cell Mol Med. 2010;14:840–860 19754673 10.1111/j.1582-4934.2009.00897.x PMC2927345

[11] Almeida IF, Valentão P, Andrade PB, et al. Oak leaf extract as topical antioxidant: free radical scavenging and iron chelating activities and in vivo skin irritation potential. Biofactors. 2008;33:267–279 19509462 10.1002/biof.5520330403

[12] Gomes-Rochette NF, Da Silveira Vasconcelos M, Nabavi SM, et al. Fruit as potent natural antioxidants and their biological effects. Curr Pharm Biotechnol. 2016;17:986–993 27109905 10.2174/1389201017666160425115401

[13] Reichling J, Schnitzler P, Suschke U, et al. Essential oils of aromatic plants with antibacterial, antifungal, antiviral, and cytotoxic properties an overview. Forsch Komplementmed. 2009;16:79–90 19420953 10.1159/000207196

[14] Miguel MG Antioxidant and antiinflammatory activities of essential oils: a short review. Molecules. 2010;15:9252–9287 21160452 10.3390/molecules15129252 PMC6259136

[15] Orzechowski A, Ostaszewski P, Jank M, et al. Bioactive substances of plant origin in food—impact on genomics. Reprod Nutr Dev. 2002;42:461–477 12537256 10.1051/rnd:2002037

[16] Zhao Y, Wu YZ, Wang M Bioactive substances of plant origin. In: Handbook of Food Chemistry. Cheung P, Mehta B, (eds). Springer: Heidelberg, Berlin; pp. 1–35; 2015

[17] Butler H Poucher's Perfumes, Cosmetics and Soaps, *10th* *ed* Kluwer Academic Publishers: London, 2000

[18] Aburjai T, Natsheh FM Plants used in cosmetics. Phytother Res. 2003;17:987–1000 14595575 10.1002/ptr.1363

[19] Karimi E, Oskouian E, Oskouian A, et al. Insight into the functional and medicinal properties of *Medicago sativa* (Alfalfa) leaves extract. J Med Plant Res. 2013;7:290–297

[20] Caunii A, Pribac G, Grozea I, et al. Design of optimal solvent for extraction of bio-active ingredients from six varieties of *Medicago sativa*. Chem Cent J. 2012;6:123 23098128 10.1186/1752-153X-6-123 PMC3495705

[21] Bora KS, Sharma A Phytochemical and pharmacological potential of *Medicago sativa*: a review. Pharm Biol. 2011;49:211–220 20969516 10.3109/13880209.2010.504732

[22] Rana MG, Katbamna RV, Padhya AA, et al. In vitro antioxidant and free radical scavenging studies of alcoholic extract of *Medicago sativa* L. Rom J Biol Plant Biol. 2010;55:15–22

[23] Fantini M, Benvenuto M, Masuelli L, et al. In vitro and in vivo antitumoral effects of combinations of polyphenols, or polyphenols and anticancer drugs: perspectives on cancer treatment. Int J Mol Sci. 2015;16:9236–9282 25918934 10.3390/ijms16059236 PMC4463587

[24] Yang B, Liu X, Gao Y Extraction optimization of bioactive compounds crocin, geniposide and total phenolic compounds from Gardenia Gardeniajasminoides Ellis fruits with response surface methodology. Innov Food Sci Emerg Technol. 2009;10:610–615

[25] Singleton VL, Orthofer R, Lamuela-Raventós RM Analysis of total phenols and other oxidation substrates and antioxidants by means of folin-ciocalteu reagent. Method Enzymol. 1999;299:152–178

[26] Matejić JS, Džamić AM, Mihajlov-Krstev TM, et al. Total phenolic and flavonoid content, antioxidant and antimicrobial activity of extracts from *Tordylium maximum*. J Appl Pharm Sci. 2013;3:55–59

[27] Brand-Williams W, Cuvelier ME, Berset C Use of a free radical method to evaluate antioxidant activity. LWT Food Sci Technol. 1995;28:25–30

[28] Borenfreund E, Puerner JA Toxicity determined in vitro by morphological alterations and neutral red absorption. Toxicol Lett. 1985;24:119–124 3983963 10.1016/0378-4274(85)90046-3

[29] Dikalov SI, Harrison DG Methods for detection of mitochondrial and cellular reactive oxygen species. Antioxid Redox Signal. 2014;20:372–382 22978713 10.1089/ars.2012.4886 PMC3887411

[30] Velioglu YS, Mazza G, Gao L, et al. Antioxidant activity and total phenolics in selected fruits, vegetables, and grain products. J Agric Food Chem. 1998;46:4113–4117

[31] Halliwell B, Gutteridge JMC Free Radicals in Biology and Medicine, *3rd* *ed* Oxford University Press: Oxford, 1999

[32] Alam MN, Bristi NJ, Rafiquzzaman M Review on in vivo and in vitro methods evaluation of antioxidant activity. Saudi Pharm J. 2013;21:143–152 24936134 10.1016/j.jsps.2012.05.002 PMC4052538

[33] Fukumoto LR, Mazza G Assessing antioxidant and prooxidant activities of phenolic compounds. J Agric Food Chem. 2000;48:3597–3604 10956156 10.1021/jf000220w

[34] Xue-Gui L, Ming-Yuan H, Pin-Yi G, et al. Bioactive constituents from *Medicago sativa* L. with antioxidant, neuroprotective and acetylcholinesterase inhibitory activities. J Funct Foods. 2018;415:371–380

[35] Kudan SB, Anupam S In vitro antioxidant and free radical scavenging potential of *Medicago sativa* Linn. J Pharm Res. 2010;3:1206–1210

[36] Zhao WS, Zhang YQ, Ren LJ, et al. Immunopotentiating effects of polysaccharides isolated from *Medicago sativa* L. (Abstract). Chung Kuo Yao Li Hsueh Pao. 1993;14:273–276 8237410

[37] Gatouillat G, Magid AA, Bertin E, et al. Cytotoxicity and apoptosis induced by alfalfa (*Medicago sativa*) leaf extracts in sensitive and multidrug-resistant tumor cells. Nutr Cancer. 2014;66:483–491 24628411 10.1080/01635581.2014.884228

[38] Alfadda AA, Sallam RM Reactive oxygen species in health and disease. J Biomed Biotechnol. 2012;2012:936486 22927725 10.1155/2012/936486 PMC3424049

